# Overexpression of Arabidopsis *BBX11* confers enhanced cold tolerance and alters stress-responsive transcriptional networks

**DOI:** 10.5511/plantbiotechnology.26.0109a

**Published:** 2026-06-25

**Authors:** Hong-Yun Chen, Benyapa Kitwetch, Wen-Jin Wang, Kai-Lun Yeh, Ya-Yu Peng, Ichiro Tarashima, Chuan-Ming Yeh

**Affiliations:** 1 Institute of Molecular Biology, National Chung Hsing University, Taichung 40227, Taiwan; 2 Interdisciplinary Program in Biotechnology, Graduate School, Chiang Mai University, Chiang Mai 50200, Thailand; 3 Advanced Plant and Food Crop Biotechnology Center, National Chung Hsing University, Taichung 40227, Taiwan; 4 Biomanufacturing and Process Research Center, National Institute of Advanced Industrial Science and Technology (AIST), Tsukuba, Ibaraki 305-8566, Japan

**Keywords:** BBX, cold, stress, transcription factor, transcriptome

## Abstract

B-box (BBX) transcription factors play critical roles in plant acclimation to environmental stresses, yet their involvement in cold stress responses remains incompletely understood. Following a genome-wide analysis of *BBX* gene expression profiles in *Arabidopsis thaliana*, we focused on *AtBBX11*, whose transcript levels increased by up to 80-fold in shoots after 24 h of cold treatment. Co-expression analysis revealed that *AtBBX11* is associated with key cold-responsive genes and stress-related pathways, including responses to cold, abscisic acid (ABA), water deprivation, and hypoxia. Functional characterization using overexpression lines demonstrated that *AtBBX11* enhances chilling tolerance, as transgenic plants exhibited significantly less reduction in shoot fresh weight compared to wild type under two chilling regimes. Transcriptome analysis under cold stress identified numerous differentially expressed genes, including selected transcription factors from several families, with *NAC* and *bHLH* being the most represented. Gene Ontology enrichment highlighted processes related to hypoxia response, phosphate starvation, and ABA signaling. Several transcription factors and hypoxia-responsive genes were further validated by Reverse Transcription-quantitative Polymerase Chain Reaction (RT-qPCR), confirming RNA-seq reliability. Collectively, these findings establish *AtBBX11* as a cold-responsive regulator that may modulate multiple stress-related pathways, providing new insights into *BBX*-mediated mechanisms underlying plant cold tolerance.

## Introduction

Climate change is a major factor affecting agriculture, as it leads to more frequent extreme weather events and unstable growing conditions that threaten global crop production. The expansion of the world population has intensified concerns regarding food availability and security. Along with the rising demand for food, plants are increasingly challenged by abiotic stresses such as drought, salinity, nutrient deficiency, and temperature extremes (Kopecká et al. 2023). Abiotic stresses strongly influence plant performance at both physiological and cellular levels by disrupting plant growth and development, reducing crop yield, and restricting the areas suitable for cultivation (Terán et al. 2024).

Among these environmental challenges, cold stress is one of the major limiting factors for crop productivity and geographic distribution. Cold stress can be categorized into two forms: chilling (0–20°C) and freezing (<0°C). It disrupts morphological and physiological processes, thereby reducing vegetative growth by limiting seed germination (Sharifi 2010). Roots are highly sensitive to low temperature, leading to impaired nutrient uptake, including nitrogen (N), phosphorus (P), and potassium (K) (Yan et al. 2012). Furthermore, the reproductive stage is the most vulnerable phase, as cold stress inhibits pollination and fertilization, resulting in poor seed set. At the physiological level, cold stress decreases photosynthetic efficiency and induces the excessive accumulation of reactive oxygen species (ROS), which cause oxidative damage (Liu et al. 2018). Chloroplasts are the primary sites of ROS generation under cold conditions, and ROS such as hydrogen peroxide (H_2_O_2_) play dual roles as damaging agents and signaling molecules. Elevated ROS can enhance abscisic acid (ABA) accumulation, which activates defense mechanisms by upregulating the expression of cold-responsive genes (Wang et al. 2018). Plant sensitivity to cold stress is therefore closely associated with ABA signaling, where chilling stress induces ABA-responsive genes that interact with transcription factors to regulate stress-responsive pathways (Chinnusamy et al. 2007). Many important crops such as rice, maize, soybean, and tomato are highly sensitive to low temperatures, highlighting the need to better understand plant cold stress responses and to identify genetic factors that improve cold tolerance (Soualiou et al. 2022).

Plants employ complex strategies to cope with cold stress, such as transcriptional reprogramming and activation of stress-responsive genes. Transcription factors play a central role by regulating downstream networks that coordinate physiological and biochemical responses (Kazemi-Shahandashti and Maali-Amiri 2018). Among them, B-box (BBX) proteins form a zinc-finger transcription factor family involved in diverse developmental processes, including seed germination, photomorphogenesis, flowering, circadian rhythm, and hormone signaling (Cao et al. 2023; Gangappa and Botto 2014). Beyond these developmental roles, BBX proteins also participate in abiotic stress responses—including ultraviolet-B (UV-B) radiation, drought, salinity, osmotic stress, heat, and cold—acting as integrators of developmental and environmental signals (Bandara et al. 2022; Gangappa and Botto 2014; Jiang et al. 2025). Evidence for *BBX* involvement in cold stress comes from multiple species: *SlBBX17* in tomato enhances tolerance via C-repeat binding factor (CBF) pathway activation (Song et al. 2023), *CmBBX24* in chrysanthemum improves cold resistance through gibberellin regulation (Yang et al. 2014), and grapevine *ZFPL* (*Zinc finger protein-like*), an Arabidopsis *BBX32* ortholog, confers cold tolerance when overexpressed (Takuhara et al. 2011). Some BBX proteins also mediate other stresses; for example, *CmBBX24* confers drought resistance (Yang et al. 2014), and *BBX24* (*SALT TOLERANCE*, *STO*) enhances salt tolerance in Arabidopsis (Nagaoka and Takano 2003).

Despite increasing evidence for the involvement of *BBX* transcription factors in abiotic stress responses, the regulatory mechanisms underlying their roles in cold stress remain poorly understood. Among *BBX* family members, *AtBBX11* has been reported as cold-inducible, yet its functional significance and downstream regulatory networks have not been characterized (Bandara et al. 2022; Gangappa and Botto 2014). In this study, we performed a genome-wide expression analysis of *BBX* genes in *Arabidopsis thaliana* and subsequently focused on *AtBBX11* for further functional characterization. To elucidate its role in cold stress acclimation, we combined bioinformatic analyses, gene co-expression networks, and transcriptome profiling with functional assays using *AtBBX11* overexpression lines. Our findings demonstrate that *AtBBX11* enhances chilling tolerance and modulates multiple stress-related pathways, including those associated with hypoxia and ABA signaling. This work provides new insights into *BBX*-mediated transcriptional regulation under cold stress and highlights the potential of *AtBBX11* as a genetic resource for improving stress tolerance in crops.

## Materials and methods

### Plant material and growth condition

The *Arabidopsis thaliana* ecotype Columbia-0 was used as the wild-type (WT) model plant in this study. Arabidopsis seeds were surface sterilized with 5.25% sodium hypochlorite (NaOCl) containing 0.025% Tween-20 for 8 min, followed by five washes with sterile double-distilled water. To break seed dormancy, sterilized seeds were suspended in 0.1% agar and incubated at 4°C for 2 days. After stratification, the seeds were sown on solid medium containing half-strength Murashige and Skoog (1/2 MS) basal salts, 0.5% (w/v) sucrose, 0.05% (w/v) MES, and 0.7% (w/v) agar (pH 5.7). Plants were grown in a controlled growth chamber at 22°C under a 16-h light/8-h dark photoperiod.

To investigate the role of Arabidopsis *BBX11*, the coding sequence of *AtBBX11* was cloned into the pBI121 vector under the control of the *CaMV 35S* promoter for constitutive overexpression. The construct was delivered to the Plant Transformation Core Facility, Academia Sinica, Taipei, Taiwan for Agrobacterium-mediated transformation using the floral dip method. Seeds from the first generation of transgenic plants (T1) were surface sterilized with 0.5% NaOCl and sown on medium containing kanamycin and timentin. Kanamycin was used to select plants carrying the transgene, while timentin inhibited Agrobacterium growth. Putative transgenic seedlings were transferred to soil, grown to maturity, and harvested for T2 seeds. The T2 progeny were screened on kanamycin-containing medium to eliminate wild-type plants, and homozygous T3 seeds were subsequently obtained for functional assays.

Based on preliminary screening and Arabidopsis eFP Browser analysis (Kilian et al. 2007; Winter et al. 2007), *AtBBX11* expression was found to be induced by cold stress. Therefore, functional analyses were performed using *CaMV 35S*-driven *AtBBX11* overexpression lines and WT plants. Seeds were germinated on 1/2 MS medium supplemented with 0.5% (w/v) sucrose, 0.05% (w/v) MES, and 0.7% (w/v) agar (pH 5.7), and grown under a 16-h light/8-h dark photoperiod at 22°C. For cold treatment, plants were exposed to 4°C for different durations.

### RNA extraction

Total RNA was extracted from Arabidopsis using the Gene-Spin Total RNA Purification Kit (PROTECH, Taiwan). Briefly, fresh seedlings rapidly frozen in liquid nitrogen and ground in a pre-cooled mortar and pestle with 450 µl of RNA Lysis/β-ME solution. The lysate was incubated at 60°C for 3 min and centrifuged at 13,000 rpm for 3 min at 4°C. The supernatant was collected, mixed with 225 µl of absolute ethanol, loaded onto the purification column, and centrifuged at 13,000 rpm for 1 min at 4°C. The column was washed with 500 µl of RNA Wash Solution I and centrifuged under the same conditions, followed by treatment with 41 µl of DNase I Mix Buffer to remove genomic DNA, and incubated at room temperature for 15 min. The column was subsequently washed with 500 µl of RNA Wash Solution I and 600 µl of RNA Wash Solution II, with centrifugation after each wash. After discarding the flow-through, the column was dried by centrifugation. RNA was eluted with 26 µl of diethyl pyrocarbonate (DEPC)-treated water. The purity of RNA was assessed by spectrophotometers (A260/A280 and A260/A230) and samples were stored at −80°C for further experiments.

### Real-Time quantitative Polymerase Chain Reaction

A total of 500 ng of RNA was used for cDNA synthesis with the Reverse Transcription Kit (SMOBIO, Taiwan). The reaction mixture contained 500 ng of total RNA, 10 mM dNTPs, 50 µM oligo dT primer, and DEPC-treated water. The mixture was incubated at 70°C for 5 min and cooled at 4°C for 1 min. Subsequently, 5X RT buffer, RNase inhibitor, reverse transcriptase, and DEPC-treated water were added, followed by incubation at 42°C for 50 min and 85°C for 5 min. The synthesized cDNA was stored at −20°C until use.

Gene expression was analyzed using the StepOnePlusTM Real-Time PCR System with SYBR Green detection. *AtTIP41* was used as the internal control gene. Each qPCR reaction mixture contained 2X SYBR Green Mix, 10 µM primers, 1 : 5 diluted cDNA, and DEPC-treated water. The thermal cycling program consisted of an initial denaturation at 95°C for 10 min, followed by 40 cycles of 95°C for 15 s and 60°C for 1 min, and a melt curve analysis (95°C for 15 s, 60°C for 1 min, and 95°C for 15 s). All reactions were performed in triplicate, and mean values were used to calculate relative expression levels.

### RNA sequencing

For transcriptome analysis, Arabidopsis wild-type (WT, ecotype Col-0) plants in the control group were grown under normal conditions for 10 days, whereas the treatment group was grown under the same conditions for 9 days followed by 24 h of cold treatment. Total RNA was extracted and used for library construction with the TruSeq Stranded mRNA Library Prep Kit (Illumina, San Diego, CA, USA). Following quality assessment of the raw reads, mRNA was isolated and converted into single-stranded cDNA using random primers. Second-strand synthesis was performed with dUTP substituted for dTTP, and an adenine overhang was added to the 3′ ends. Index adapters were then ligated to both termini of the cDNA, and adapter-containing fragments were enriched by PCR amplification. Library quality was assessed using an Agilent 2100 Bioanalyzer and quantified with a Real-Time PCR System. Differentially expressed genes (DEGs) were subsequently identified and subjected to functional annotation using Gene Ontology (GO) analysis and other relevant bioinformatic approaches. The RNA-seq data generated in this study have been deposited in the DNA Data Bank of Japan (DDBJ) under accession number E-GEAD-1188.

### Bioinformatics analysis

Amino acid sequences of AtBBX11 and its homologous transcription factors were retrieved from the TAIR database and aligned using Molecular Evolutionary Genetics Analysis (MEGA) software. Expression profiles of *BBX* genes under abiotic stress in shoot and root tissues were examined using the Arabidopsis eFP Browser 2.0 (Kilian et al. 2007; Winter et al. 2007). Co-expression analysis was performed with the ATTED-II database (Obayashi et al. 2022), and genes showing highly correlated expression patterns were identified as potential co-regulators or downstream targets.

### Statistical analysis

All experimental data were analyzed using Microsoft Excel for analysis of variance (ANOVA) to test significant differences among treatments. When ANOVA indicated significance, the Least Significant Difference (LSD) test was performed for multiple comparisons. Differences were represented by different lowercase letters in figures, with statistical significance defined at *p*≤0.05.

## Results

### In silico identification of cold-responsive *BBX* genes in *Arabidopsis thaliana*

Cold stress is a major factor influencing *BBX* gene regulation in *Arabidopsis thaliana*. Previous studies identified seven *AtBBX* genes—*AtBBX2*, *AtBBX6*, *AtBBX7*, *AtBBX11*, *AtBBX15*, *AtBBX24*, and *AtBBX29*—as cold-inducible (Bandara et al. 2022; Gangappa and Botto 2014). Later work further clarified the roles of previously reported *BBX* genes and identified additional ones involved in cold responses. For example, *AtBBX7* and *AtBBX8* enhance freezing tolerance by modulating cold-responsive gene expression, while *AtBBX28* and *AtBBX29* promote flowering at low temperatures (Li et al. 2021; Wang et al. 2021). However, the roles of *AtBBX2*, *AtBBX6*, *AtBBX11*, *AtBBX15*, and *AtBBX24* remain unclear.

**Figure 1:**
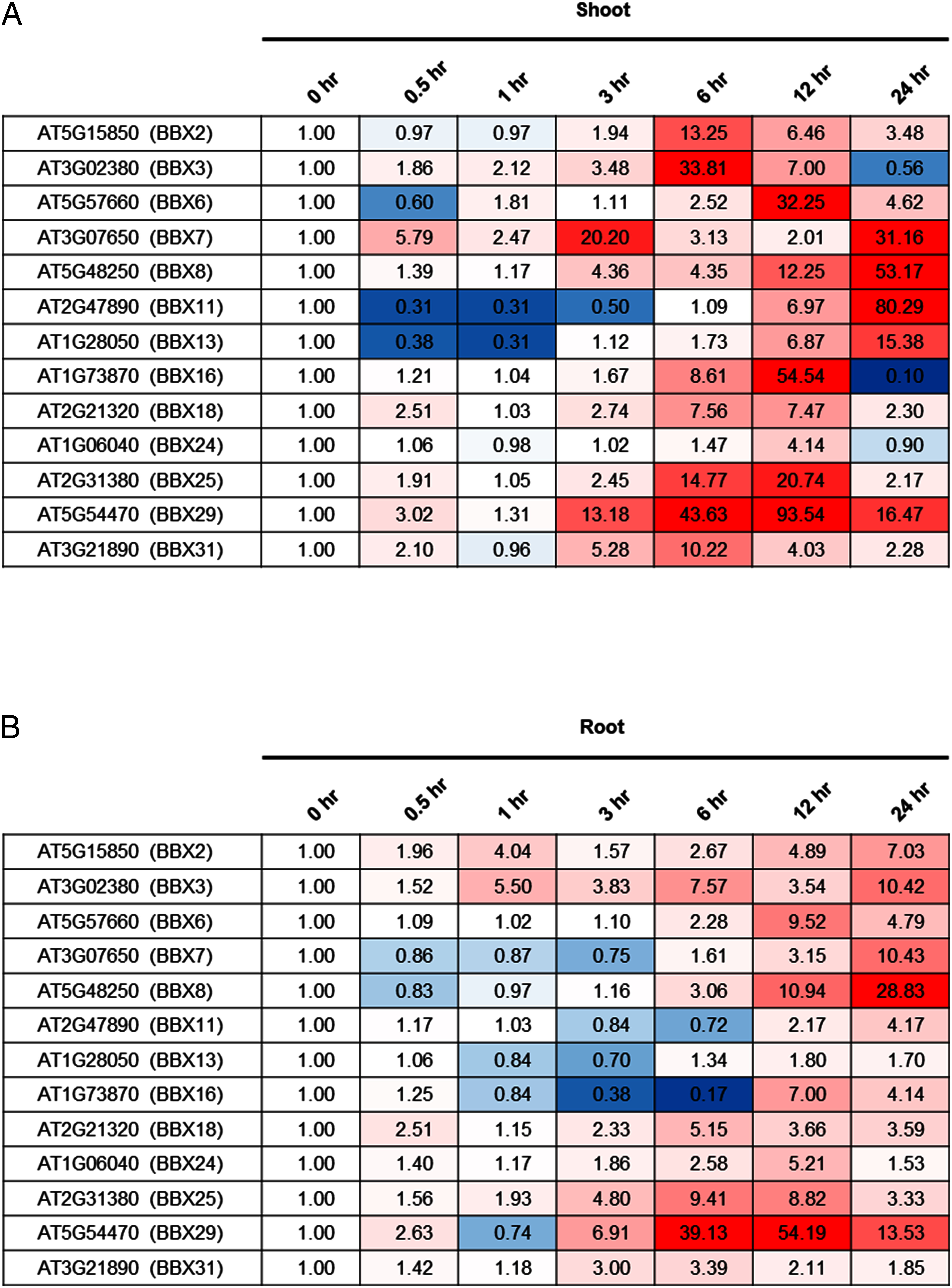
Figure 1. Heat-map representation of cold-responsive *BBX* genes in Arabidopsis. Expression data for *BBX* transcription factor genes were obtained from the Arabidopsis eFP Browser (Kilian et al. 2007; Winter et al. 2007) to assess their responsiveness to cold stress. The heat map displays relative expression levels of all *BBX* genes with available data that showed induction under cold treatment across the time course. Data were reorganized and visualized using Microsoft Excel to highlight differential expression patterns.

To address this gap, we conducted a genome-wide analysis of *AtBBX* gene expression under cold stress by examining the expression profiles of all *AtBBX* family members using the Arabidopsis eFP Browser. This publicly accessible transcriptomic database covers diverse conditions, including abiotic stress, and allowed us to identify additional *AtBBX* candidates potentially involved in cold stress responses (Kilian et al. 2007; Winter et al. 2007). Our analysis revealed that, in addition to the *AtBBX* genes previously reported in earlier studies, *AtBBX3*, *AtBBX13*, *AtBBX16*, *AtBBX18*, *AtBBX25* and *AtBBX31* also showed induction under cold treatment. However, expression data for *AtBBX10*, *AtBBX15*, *AtBBX17*, *AtBBX26*, *AtBBX28*, *AtBBX30*, and *AtBBX32* were not available in the eFP Browser and therefore could not be evaluated. To narrow down potential targets, we first excluded *AtBBX* genes that have already been functionally characterized. Among the remaining candidates, *AtBBX11* exhibited the highest level of cold-induced expression in shoots, with moderate induction in roots. Based on this stepwise selection process, we prioritized *AtBBX11* for detailed functional investigation in the context of cold stress response. Specifically, *AtBBX11* expression increased approximately 7-fold and 80-fold in shoots at 12 and 24 h of cold treatment (4°C), respectively, and 2-fold and 4-fold in roots at the same time points (Figure 1, Supplementary Table S1).

To gain deeper insight into its potential role, we performed a gene co-expression analysis using the Arabidopsis gene co-expression database ATTED-II (Obayashi et al. 2022). Several genes co-expressed with *AtBBX11* are well-known for their involvement in cold stress responses, including key transcription factors such as *DREB2C*, *DREB2A*, and *DREB1A* (*CBF3*), as well as cold-responsive genes such as *COR47*, *COR413-PM1*, *COR15B*, *COR78* (*RD29A*), *COR6.6*, and *COR15A* (Supplementary Table S2). Additionally, we explored the biological relevance of *AtBBX11*-associated genes by retrieving the top 200, 300, and 500 co-expressed genes from ATTED-II and performed Gene Ontology (GO) enrichment analysis. The results revealed that these co-expressed genes are predominantly associated with abiotic stress responses, including response to cold, abscisic acid, water deprivation, hypoxia, heat, and oxidative stress (Supplementary Figure S1). Among these, genes related to cold stress were the most enriched. The presence of these cold-related genes in the *AtBBX11* co-expression network provides additional support for the hypothesis that *AtBBX11* may play a functional role in the regulation of cold stress responses in Arabidopsis.

### Experimental validation of *AtBBX11* as a cold-responsive gene in *Arabidopsis thaliana*

**Figure 2:**
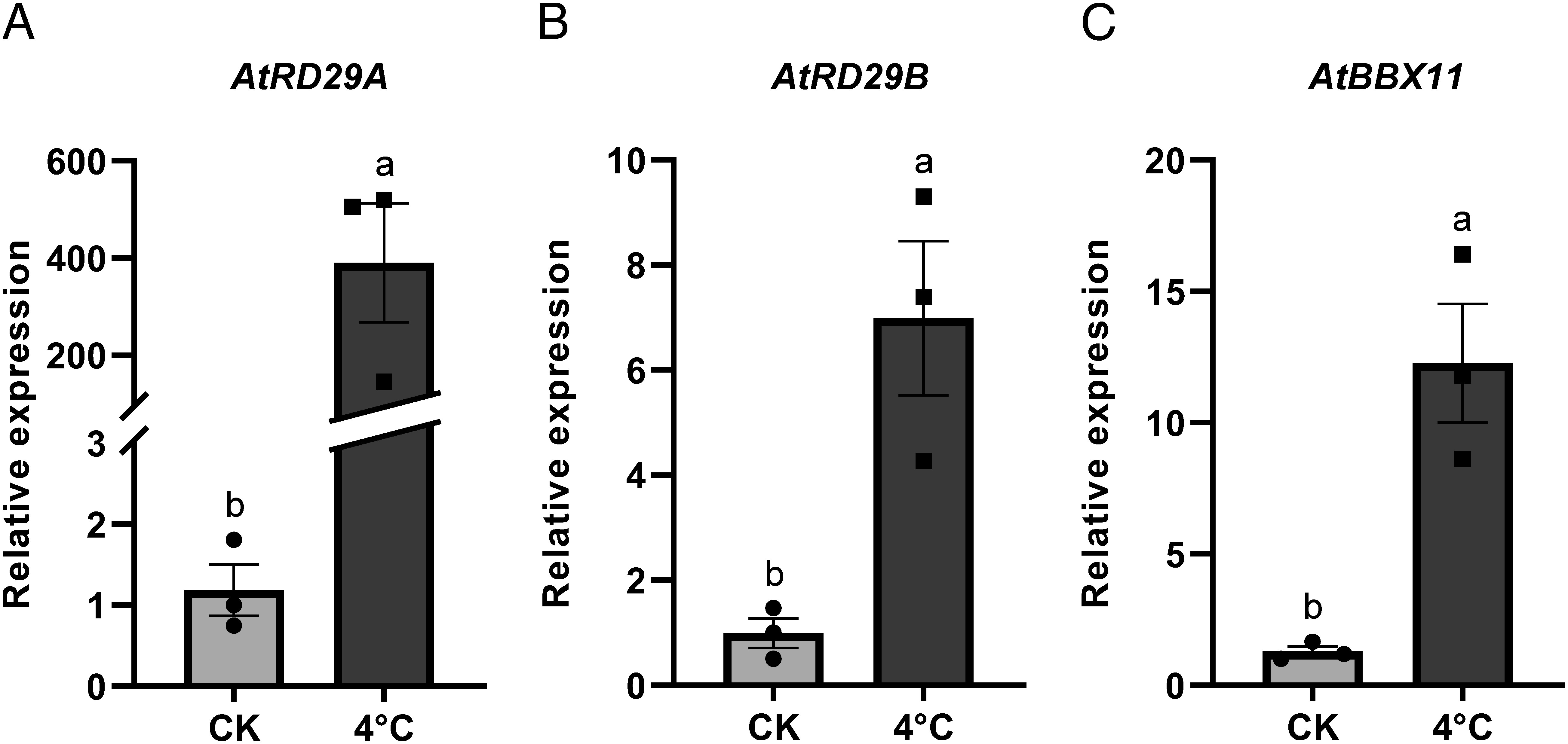
Figure 2. Expression of cold-responsive marker genes and *AtBBX11* in wild-type (WT) Arabidopsis under cold treatment. Plants were grown on 1/2 MS solid medium at 22°C for 9 days and then transferred to 4°C for 1 day. Relative expression levels of the cold-responsive marker genes *RD29A* (A) and *RD29B* (B), and *AtBBX11* (C), were analyzed by RT-qPCR. Gene expression levels were normalized to the reference gene and are presented as fold changes relative to the control treatment. Data represent the mean±SD. of three biological replicates (*n*=3). Different lowercase letters above the bars indicate statistically significant differences among treatments, as determined by one-way analysis of variance (ANOVA) followed by an LSD test (*p*≤0.05). Primer sequences used for RT-qPCR are listed in Supplementary Table S3.

To validate the cold-induced expression of *AtBBX11* suggested by eFP Browser data—which showed its strongest induction after 24 h of cold treatment—we exposed *Arabidopsis thaliana* seedlings to cold for 24 h. Seedlings were grown on 1/2 MS medium at 22°C for 9 days, then transferred to 4°C for 24 h before RNA extraction for RT-qPCR analysis. To confirm the effectiveness of our cold treatment, we examined two well-characterized cold-inducible marker genes, *RD29A* and *RD29B* (Kim S and Kim W 2013; Kwak et al. 2005). Both showed strong induction under cold stress, validating our protocol (Figure 2). Under the same conditions, *AtBBX11* transcript levels were significantly upregulated, consistent with eFP Browser. Our RT-qPCR analysis confirmed the strong induction of *AtBBX11* under cold stress, providing experimental evidence for its potential role in cold stress regulation in Arabidopsis.

### Overexpression of *AtBBX11* enhances cold stress tolerance in Arabidopsis

**Figure 3:**
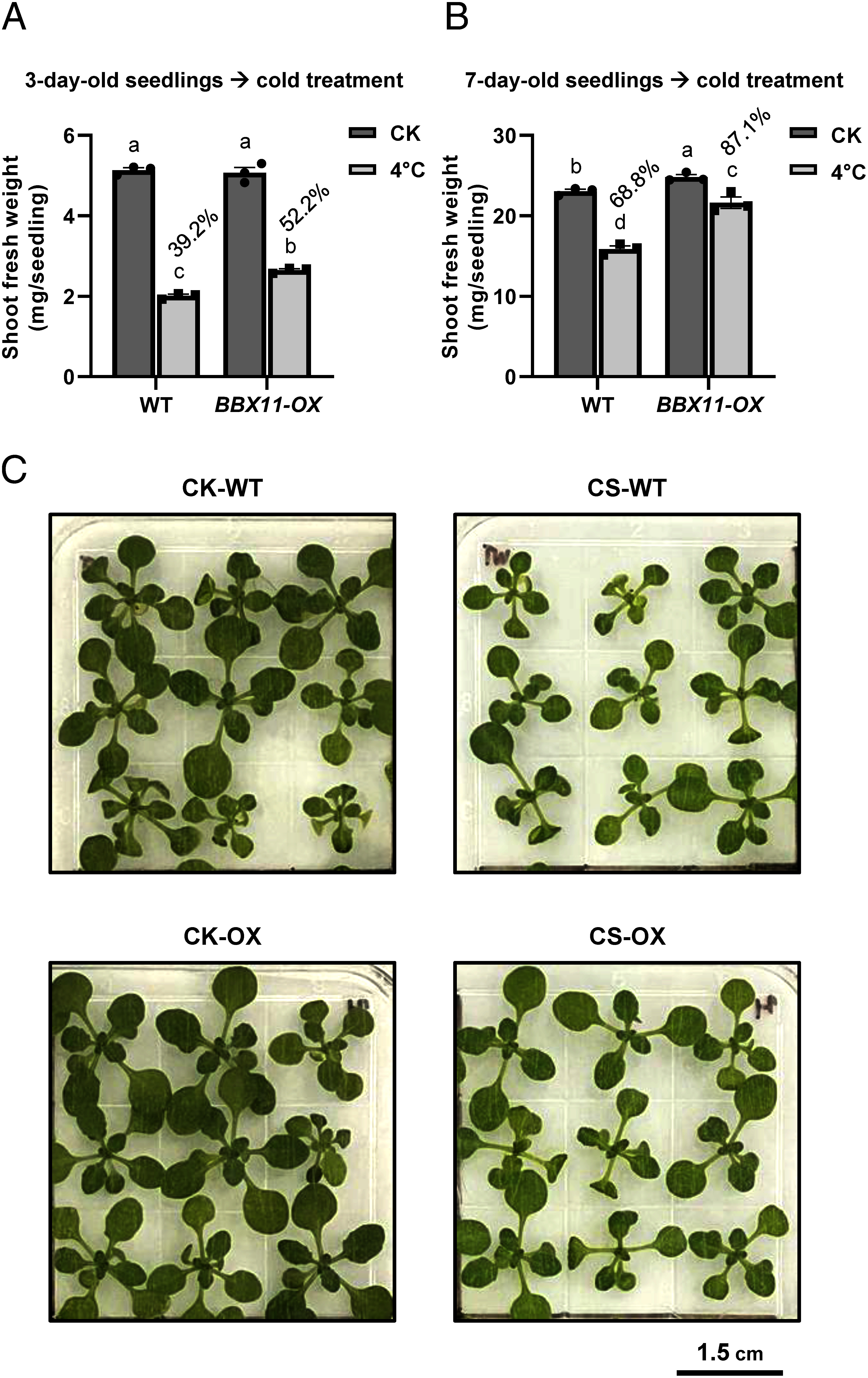
Figure 3. Shoot fresh weight and phenotypes of wild-type (WT) and *AtBBX11*-overexpressing (*AtBBX11*-OX). Plants were grown on 1/2 MS solid medium at 22°C for 3 days, transferred to 4°C for 3 days, and then returned to 22°C for 4 days (A). Alternatively, plants were grown at 22°C for 7 days, followed by transfer to 4°C for 3 days and recovery at 22°C for 4 days (B). Shoot fresh weight was measured after each treatment. (C) Representative images of WT and *AtBBX11-OX* plants under control (CK) and cold stress (CS) conditions following the treatment described in (B). Values represent the mean±SD of three biological replicates (*n*=3), and different letters indicate significant differences (*p*≤0.05, two-way ANOVA followed by LSD test).

To assess whether *AtBBX11* contributes to cold stress tolerance, we generated *AtBBX11* overexpression (*AtBBX11*-OX) transgenic lines and compared their performance with wild-type (WT) plants under two different chilling conditions. For condition A, plants were grown on 1/2 MS solid medium at 22°C for 3 days, transferred to 4°C for 3 days, and then returned to 22°C for 4 days before shoot fresh weight was measured (Figure 3A). Under this treatment, shoot fresh weight decreased by 60.8% in WT and 47.8% in *AtBBX11*-OX plants relative to the control group. For condition B, plants were grown at 22°C for 7 days, transferred to 4°C for 3 days, and then returned to 22°C for 4 days before measurement (Figure 3B, C). Under this treatment, shoot fresh weight decreased by 31.2% in WT and 12.9% in *AtBBX11*-OX plants relative to the control group. These results suggest that *AtBBX11* overexpression may confer improved tolerance to chilling stress, although further studies are needed to fully elucidate its role.

### Expression analysis of cold-responsive genes in *AtBBX11* overexpression lines

**Figure 4:**
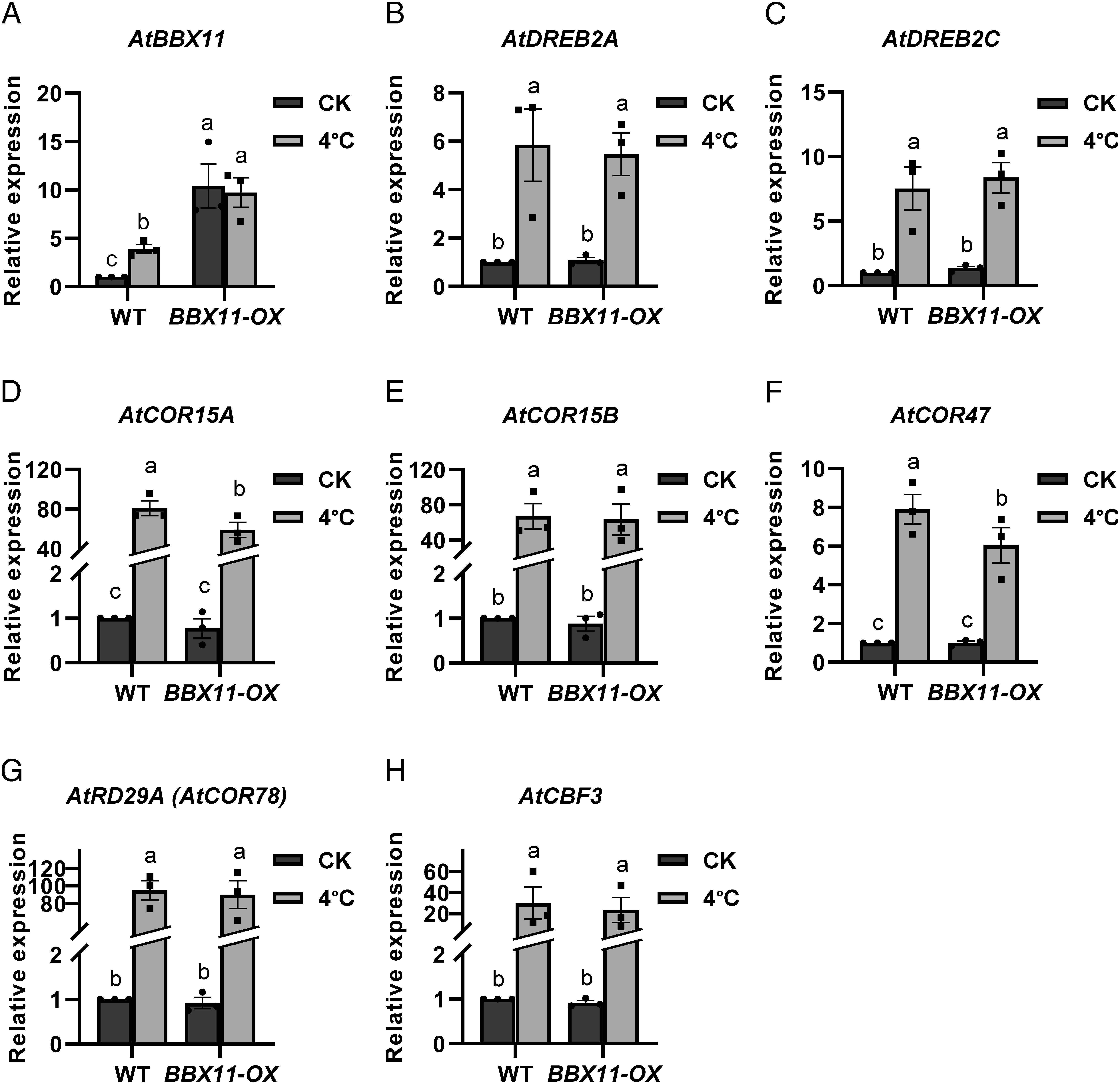
Figure 4. Expression profiles of cold-responsive genes in WT and *AtBBX11*-OX plants under cold treatment. Plants were grown on 1/2 MS solid medium at 22°C for 9 days, then transferred to 4°C for 1 day. Total RNA was extracted and analyzed by RT-qPCR to assess the expression levels. Genes: A, *AtBBX11*; B, *DREB2A*; C, *DREB2C*; D, *COR15A*; E, *COR15B*; F, *COR47*; G, *RD29A*; H, *CBF3*. Values represent means of three replicates (*n*=3). Different letters indicate significant differences (*p*≤0.05, two-way ANOVA, LSD test). Primer sequences used for RT-qPCR are listed in Supplementary Table S3.

Based on the ATTED-II gene co-expression analysis, AtBBX11 appears to be associated with cold-responsive genes such as *DREBs*, *CORs*, and *CBF3*, which are central regulators of cold stress responses (Supplementary Table S2). To identify potential downstream genes and signaling pathways regulated by AtBBX11, we first confirmed successful overexpression by showing that *AtBBX11* expression was consistently higher in OX lines than in WT under both control and cold stress conditions (Figure 4A). After confirming this, we examined whether the expression of co-expressed cold-responsive genes (*DREB2A*, *DREB2C*, *COR15A*, *COR15B*, *COR47*, *RD29A*, and *CBF3*) was altered in *AtBBX11* overexpression lines. Under control conditions, all genes displayed similar basal expression levels in both genotypes. In contrast, these genes were strongly induced by cold stress in both WT and *AtBBX11-OX* plants. However, no significant differences were observed between the two genotypes (Figure 4B–H). These results indicate that *DREB2A*, *DREB2C*, *COR15A*, *COR15B*, *COR47*, *RD29A*, and *CBF3* are not directly regulated downstream of AtBBX11. Nonetheless, the strong induction of these genes confirms that the cold treatments were effective.

### Overview of DEGs identified from transcriptome analysis

To investigate the molecular basis underlying the enhanced cold tolerance observed in *AtBBX11*-OX plants, we performed transcriptome analysis under cold stress, with growth conditions and RNA extraction procedure identical to those used for RT-qPCR. The resulting datasets were subjected to GO enrichment analyses to gain insight into the potential downstream targets and signaling networks regulated by AtBBX11. Differentially expressed genes (DEGs) were identified based on defined statistical thresholds. Genes exhibiting a fold change ≥2 with *p*≤0.05 were classified as upregulated, whereas those with a fold change ≤0.5 and *p*≤0.05 were designated as downregulated.

A comparative transcriptomic analysis was performed to identify DEGs among the four pairwise comparisons: transgenic lines versus wild-type under control conditions (CK-OX/CK-WT), transgenic lines versus wild-type under cold stress (CS-OX/CS-WT), wild-type under cold stress versus control (CS-WT/CK-WT), and transgenic lines under cold stress versus control (CS-OX/CK-OX). The Venn diagram revealed both shared and distinct transcriptional responses across genotypes and treatments.

**Figure 5:**
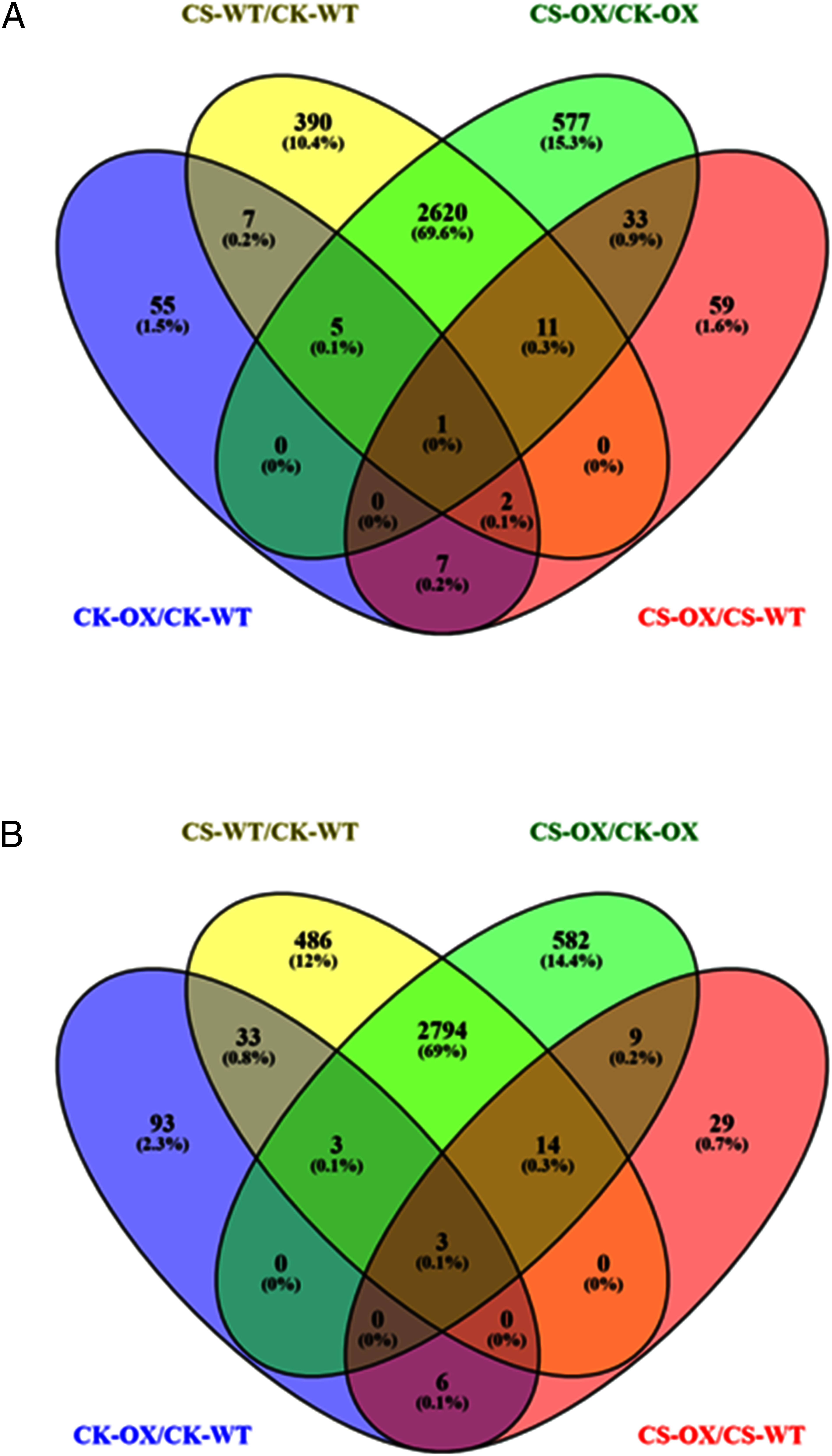
Figure 5. Venn diagrams of differentially expressed genes in *AtBBX11* overexpression lines and wild-type Arabidopsis under cold stress. Wild-type and *AtBBX11*-OX seedlings were grown on 1/2 MS solid medium at 22°C for 9 days, then transferred to 4°C for 1 day. Shoots were harvested immediately after cold treatment, and total RNA was extracted for transcriptome sequencing. Four pairwise comparisons were performed: CK-OX/CK-WT, CS-WT/CK-WT, CS-OX/CK-OX, and CS-OX/CS-WT. DEGs were identified and visualized using Venn diagrams generated with Venny 2.1 [https://bioinfogp.cnb.csic.es/tools/venny/ (Accessed Dec 16, 2025)]. Panel (A) shows up-regulated DEGs (fold change ≥2, *p*≤0.05), and Panel (B) shows down-regulated DEGs (fold change ≤0.5, *p*≤0.05). Gene counts and percentages are indicated in each intersection.

For upregulated genes, 77 DEGs were detected in CK-OX/CK-WT, 113 in CS-OX/CS-WT, 3,036 in CS-WT/CK-WT, and 3,247 in CS-OX/CK-OX (Figure 5A). Only one gene, At2g20800 (*NDB4*), was commonly upregulated across all four comparisons, indicating a core gene responsive to both cold stress and *AtBBX11* overexpression. For downregulated genes, 138 DEGs were identified in CK-OX/CK-WT, 61 in CS-OX/CS-WT, 3,333 in CS-WT/CK-WT, and 3,405 in CS-OX/CK-OX (Figure 5B). Only three genes, AT2G37720 (*TBL15*), AT1G52040 (*MBP1*), and AT2G15042 (*Leucine-rich repeat family protein*), were commonly downregulated across all comparisons. Together, these transcriptomic patterns highlight both shared and genotype-specific gene expression signatures, emphasizing the extensive transcriptional reprogramming associated with cold stress and the regulatory impact of *AtBBX11* overexpression.

### GO biological process enrichment analysis

To clarify the functional implications of these transcriptional changes, we performed GO Biological Process (BP) enrichment analysis with two main objectives: (1) to examine whether *AtBBX11* overexpression under cold stress (CS-OX/CK-OX) triggers biological pathways that are absent in WT (CS-WT/CK-WT) or become more prominently enriched compared to WT, and (2) to determine whether *AtBBX11* overexpression under cold stress (CS-OX/CK-OX) influences the same pathways as WT (CS-WT/CK-WT) but alters the expression of specific genes within those pathways, either by upregulating or downregulating their expression.

Our analysis revealed that, under cold stress, upregulated DEGs from WT (CS-WT/CK-WT) and *AtBBX11*-OX (CS-OX/CK-OX) comparisons exhibited highly similar enrichment patterns, with the top 14 pathways overlapping between the two genotypes. These included regulation of DNA-templated transcription, response to abscisic acid, response to cold, RNA modification, and embryo development ending in seed dormancy (Supplementary Figure S2A). For downregulated DEGs, both WT (CS-WT/CK-WT) and *AtBBX11*-OX (CS-OX/CK-OX) plants also displayed comparable enrichment profiles, with highly ranked categories such as regulation of DNA-templated transcription, defense response, protein ubiquitination, cellular response to hypoxia, and defense response to pathogens (Supplementary Figure S2B).

Together with the Venn diagram analysis, these findings indicate that WT and *AtBBX11*-OX plants respond to cold stress through largely overlapping biological processes. Therefore, the improved cold tolerance observed in OX plants is unlikely due to the activation of entirely new pathways, but rather to differences within shared pathways—such as the number of genes involved or their expression levels in OX plants.

**Figure 6:**
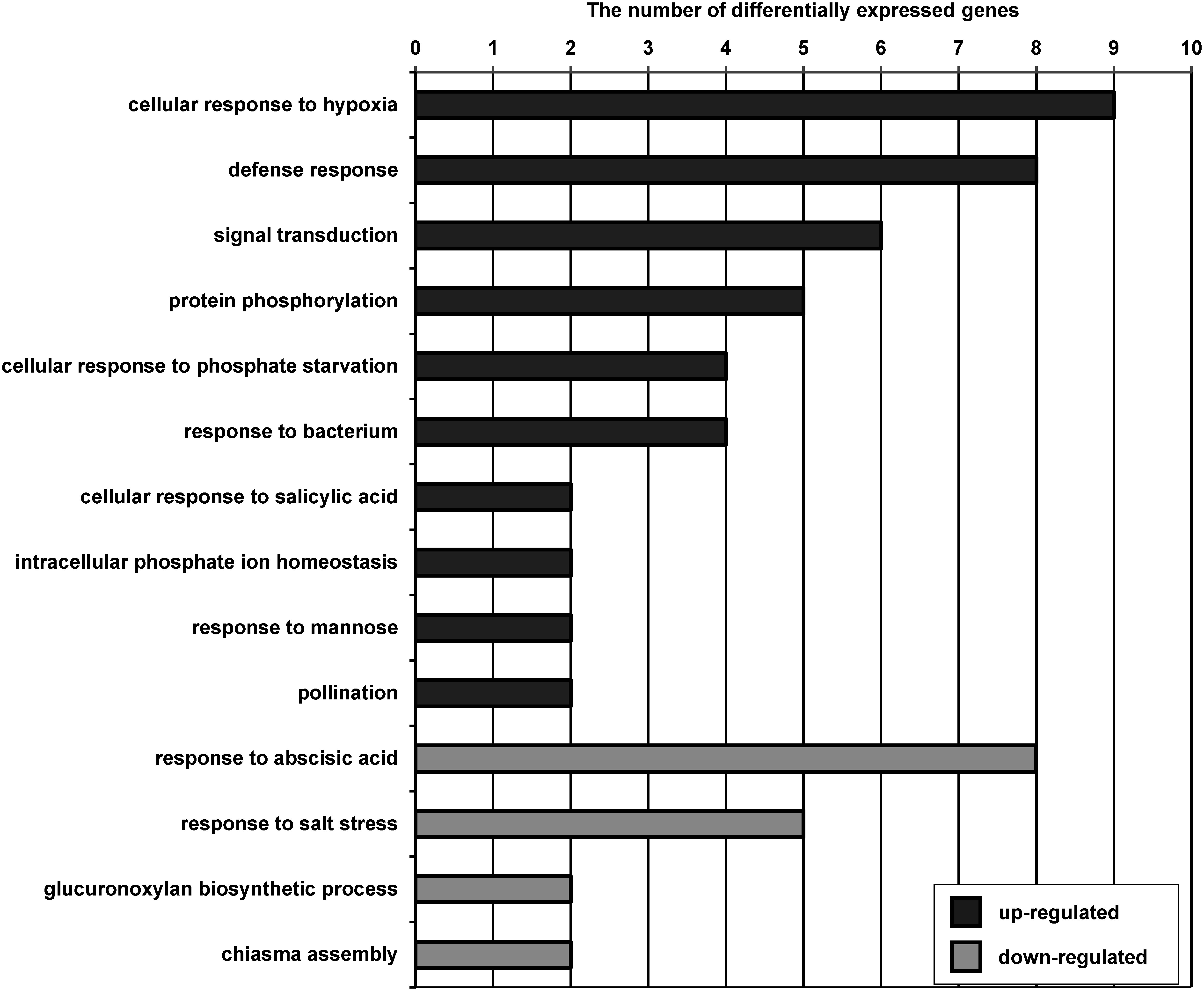
Figure 6. GO-BP enrichment analysis of DEGs between *AtBBX11*-OX and wild-type Arabidopsis under cold stress. Transcriptomes of *AtBBX11*-OX and wild-type plants under cold treatment (CS-OX/CS-WT) were compared, and both up-regulated and down-regulated genes were analyzed for GO-BP enrichment. Dark bars indicate up-regulated genes, and light gray bars indicate down-regulated genes, as shown in the legend.

To further investigate the potential role of *AtBBX11* under cold stress, we compared the transcriptomes of *AtBBX11*-overexpressing plants and wild-type Arabidopsis under cold treatment (CS-OX/CS-WT). GO-BP enrichment analysis was conducted on DEGs identified in the transgenic plants under cold stress. Because relatively few GO-BP terms were obtained under the initial criteria, we broadened the selection threshold for DEGs: genes with a fold change ≥1.5 and *p*≤0.05 were defined as upregulated, whereas those with a fold change ≤0.67 and *p*≤0.05 were considered downregulated. The analysis revealed that upregulated genes were primarily associated with cellular response to hypoxia, defense response, signal transduction, protein phosphorylation, and cellular response to phosphate starvation (Figure 6). Interestingly, when we performed a similar GO-BP analysis under normal conditions (CK-OX/CK-WT), cellular response to hypoxia was also ranked first among the enriched categories, suggesting that this process may represent a consistent feature of *AtBBX11* overexpression rather than a condition-specific response. However, because only 77 upregulated DEGs were identified under CK-OX/CK-WT, the GO analysis yielded a single enriched term (cellular response to hypoxia). Therefore, the result is described in the text instead of shown in a figure. In contrast, downregulated genes were mainly related to response to abscisic acid (Figure 6).

Taken together, the GO-BP enrichment results indicate that, when considering both upregulated and downregulated genes, the affected biological processes include hypoxia response, phosphate starvation, ABA signaling, and stress response, suggesting that *AtBBX11* may be involved in signaling and regulatory mechanisms underlying plant acclimation to multiple abiotic stresses. While further functional validation is required, these observations imply that genes associated with cellular response to hypoxia could be important downstream components of *AtBBX11*-mediated pathways. Future studies will aim to characterize these genes to better understand their potential contribution to cold stress tolerance.

### Transcription factor analysis of DEGs under cold stress and normal conditions

To further explore the transcriptional regulatory role of AtBBX11 suggested by GO analysis, we focused on transcription factors among DEGs identified in the CS-OX/CS-WT comparison. We selected transcription factors showing a fold-change greater than 1.5 with *p*≤0.05 and performed bioinformatic analyses (Table 1). These analyses included determining whether the transcription factors belong to the same family and share close phylogenetic relationships (i.e., potentially functionally redundant), examining published reports on their roles in abiotic stress, and verifying their expression profiles under various abiotic stresses using the Arabidopsis eFP Browser. Under cold stress, the 11 induced transcription factors in *AtBBX11*-OX plants were distributed across several families, with *NAC* (three genes) and *bHLH* (two genes) being the most represented, while the remaining families were each represented by a single gene. Among the *NAC* family members, *NAC085* and *NAC044* are closely related to each other (Ooka et al. 2003), suggesting possible functional redundancy.

**Table 1:** Table 1. Transcription factors with the highest expression in *AtBBX11*-OX compared to WT plants under cold stress (CS-OX/CS-WT) and their abiotic stress regulation profiles.

Locus	Gene name	TF family	Fold change (CS-OX/CS-WT)	*p*-value	Publication	Abiotic stress (Shoot)	Abiotic stress (Root)	Hypoxia stress
AT4G25530	*HDG6*	HD-ZIP	18.10	0.05	—	—	—	V
AT1G10586	*bHLH168*	bHLH	17.37	0.01	NA	NA	NA	NA
AT3G44460	*bZIP67*	bZIP	13.78	0.004	—	C, D, H	H	V
AT5G06650	*GIS2*	C2H2	4.46	0.05	—	—	—	—
AT2G24681	*REM*	B3	4.30	0.05	NA	NA	NA	NA
AT5G14490	*NAC085*	NAC	3.85	0.04	—	NA	NA	NA
AT2G13960	—	MYB	2.61	0.05	—	H	C, H	—
AT5G61430	*NAC100*	NAC	2.03	0.01	—	C	H, O	—
AT3G01600	*NAC044*	NAC	1.99	0.0003	—	NA	NA	NA
AT4G24150	*GRF8*	GRF	1.70	0.05	—	C, H, O, S	D, H, O, S, W	—
AT4G11140	*CRF1*	bHLH	1.61	0.002	—	D, H, O	H	—

The Arabidopsis eFP Browser database was used to assess whether these transcription factors in shoots or roots were induced by different abiotic stresses (≥ 2-fold compared to control). Abbreviations for stresses: C, cold; D, drought; H, heat; O, osmotic; S, salt; W, wounding. “—” indicates no related published literature or no induction under abiotic stress in the eFP Browser. NA (not available/not applicable) indicates no published data or unavailable information in the eFP Browser. “V” indicates induction under hypoxia stress.

In addition, we examined transcription factors among DEGs under normal conditions (CK-OX/CK-WT). Only three transcription factors met the selection criteria (fold change ≥1.5, *p*≤0.05), and these are listed in Table 2. When comparing transcription factors between cold stress (CS-OX/CS-WT) and normal conditions (CK-OX/CK-WT), no overlapping genes were found under the defined thresholds. Notably, *NAC085* was detected under both conditions but excluded from Table 2 because its *p*-value (0.06) was slightly above the commonly used threshold of 0.05. Although this does not meet the conventional cutoff, it is important to note that a *p*-value of 0.06 does not imply a lack of biological relevance; statistical significance should not be interpreted dichotomously. The presence of *NAC085* in both conditions may still indicate a meaningful association and suggest a potential role in *AtBBX11*-mediated regulatory networks. Given their close phylogenetic relationship (as noted above), whether *NAC085*, together with *NAC044*, contributes to functional redundancy or plays a role in stress acclimation remains to be determined.

**Table 2:** Table 2. Transcription factors with the highest expression in *AtBBX11*-OX compared to WT plants under control conditions (CK-OX/CK-WT) and their abiotic stress regulation profiles.

Locus	Gene name	TF family	Fold change (CK-OX/CK-WT)	*p*-value	Publication	Abiotic stress (Shoot)	Abiotic stress (Root)	Hypoxia stress
AT3G11020	*DREB2B*	AP2-EREBP	1.75	0.027	S	C, D, H, O, S, W	C, D, H, O, S, W	—
AT1G53160	*SPL4*	SBP	1.65	0.007	—	—	O	—
AT2G40140	*CZF1*	Zinc finger	1.62	0.026	—	C, D, O, S, W	C, D, O, S	V

The Arabidopsis eFP Browser database was used to assess whether these transcription factors in shoots or roots were induced by different abiotic stresses (≥ 2-fold compared to control). Abbreviations for stresses: C, cold; D, drought; H, heat; O, osmotic; S, salt; W, wounding. “—” indicates no related published literature or no induction under abiotic stress in the eFP Browser. NA (not available/not applicable) indicates no published data or unavailable information in the eFP Browser. “V” indicates induction under hypoxia stress.

Further examination under the CS-OX/CS-WT comparison indicated that some of these transcription factors are associated with abiotic stress responses, with four genes induced by cold treatment (Table 1). However, expression data for certain transcription factors were not available in the Arabidopsis eFP Browser. Whether these cold-induced transcription factors contribute to *AtBBX11*-mediated cold tolerance warrants further investigation. These findings offer preliminary insights into transcriptional regulation associated with *AtBBX11* overexpression under cold stress.

### RT-qPCR validation of differentially expressed genes identified by transcriptome analysis

**Figure 7:**
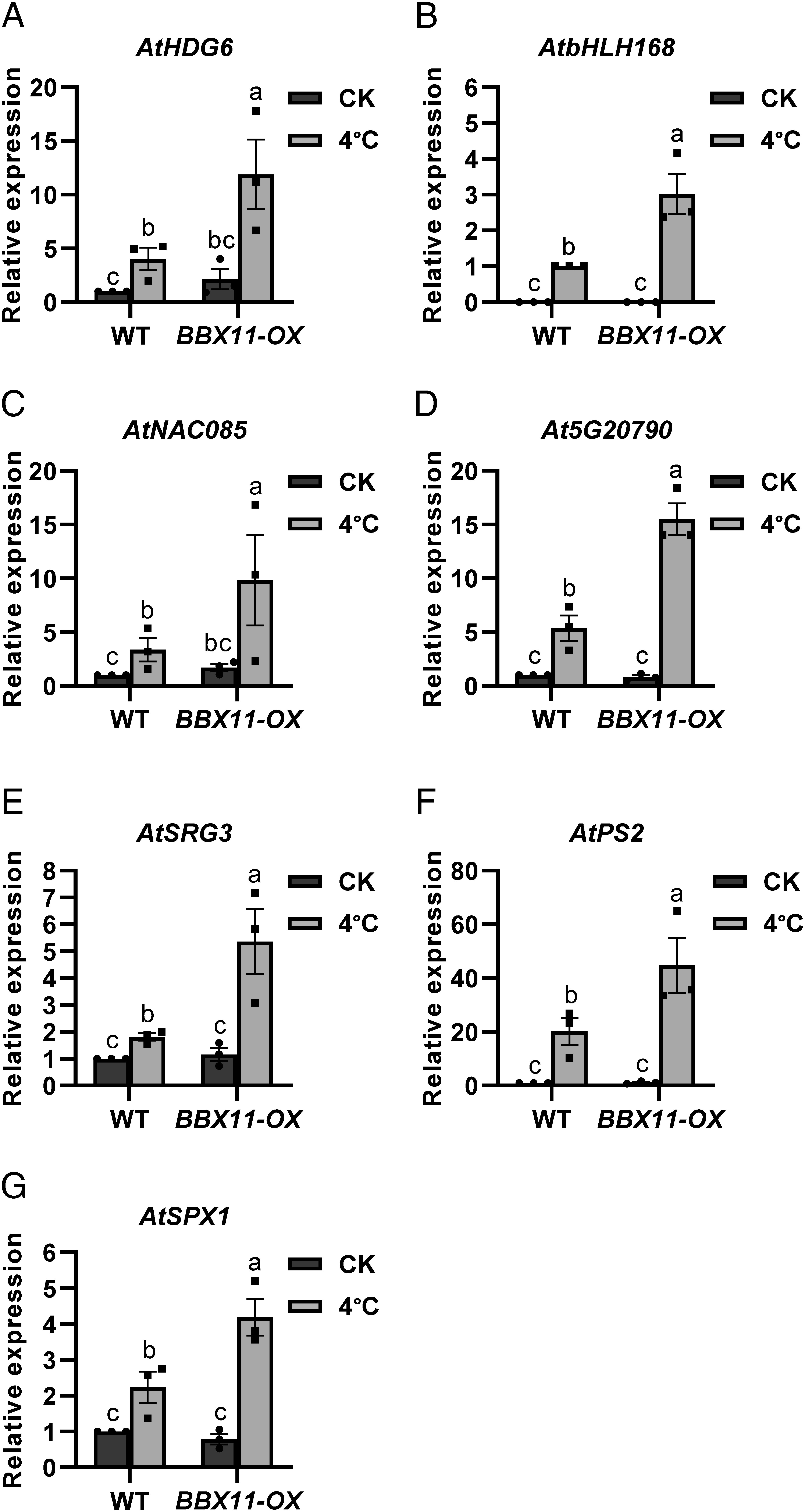
Figure 7. Validation of differentially expressed genes induced by cold stress in Arabidopsis. Arabidopsis seedlings were grown on 1/2 MS solid medium at 22°C for 9 days and then transferred to 4°C for 1 day. Total RNA was extracted and subjected to RT-qPCR to determine the transcript levels of *AtHDG6* (A), *AtbHLH168* (B), *AtNAC085* (C), *AT5G20790* (D), *AtSRG3* (E), *AtPS2* (F), and *AtSPX1* (G). Data represent the mean±standard deviation of three biological replicates (*n*=3). Statistical significance was determined using two-way ANOVA followed by LSD test; different letters denote significant differences at *p*≤0.05. Primer sequences used for RT-qPCR are listed in Supplementary Table S3.

To validate the RNA-seq results, RT-qPCR was performed on seven genes, including three transcription factors listed in Table 1 (Figure 7A–C), as well as four hypoxia-responsive genes selected based on GO analysis of DEGs, which indicated that “cellular response to hypoxia” was the most enriched category (Figure 7D–G). Consistent with this classification, analysis using the eFP Browser further showed that these genes are upregulated under hypoxia conditions (Supplementary Figure S3). These hypoxia-responsive genes include AT5G20790, which encodes a transmembrane protein; AtSRG3 (senescence-related gene 3), a member of the glycerophosphodiester phosphodiesterase (GDPD) family; AtPS2 (inorganic pyrophosphatase 1), encoding a pyrophosphate-specific phosphatase with an alkaline catalytic optimum; and AtSPX1 (SPX domain-containing protein 1). The RT-qPCR results were consistent with the transcriptome data, indicating that under cold stress, all seven genes showed higher expression in OX lines than in WT. These findings support the reliability of the RNA-seq analysis and suggest that *AtBBX11* overexpression may modulate multiple stress-related pathways, including those associated with hypoxia, providing a foundation for future functional studies.

## Discussion

Co-expression analysis using ATTED-II revealed that *AtBBX11* is associated with multiple cold-responsive genes, including *DREB2A*, *DREB2C*, *CBF3*, and *COR* family members, which are central components of the CBF-dependent signaling cascade (Supplementary Table S2). This pathway is rapidly activated under low temperatures and promotes the expression of genes that stabilize membranes, protect proteins from denaturation, and maintain osmotic balance, thereby enhancing cold tolerance (Hwarari et al. 2022; Liang et al. 2022). The strong co-expression of *AtBBX11* with these genes, together with its own induction under cold treatment, suggests that it may function within or parallel to established cold signaling networks, potentially acting as an upstream regulator or integrator of stress responses.

Functional assays demonstrated that *AtBBX11* overexpression improves chilling tolerance, supporting its positive role in cold stress acclimation (Figure 3). Structurally, AtBBX11 belongs to the type II BBX subgroup, containing two B-box domains and one CCT domain, a conserved configuration commonly associated with transcriptional regulation and the integration of multiple signaling pathways (Gangappa and Botto 2014). Transcriptome analysis and GO enrichment further revealed that AtBBX11-dependent genes are associated not only with cold response but also with hypoxia, defense response, ABA signaling, and phosphate starvation, indicating broader involvement in stress-related regulatory networks (Figure 6). This is consistent with previous reports of BBX proteins acting as integrators of environmental and hormonal signals (Gangappa and Botto 2014; Gangappa et al. 2013; Kumagai et al. 2008; Yang et al. 2014). Collectively, these findings could position *AtBBX11* as a potential hub in transcriptional networks that coordinate cold tolerance and interact with other stress pathways. Future studies should focus on identifying direct downstream targets and dissecting the molecular mechanisms by which *AtBBX11* interacts with ABA and hypoxia signaling to enhance stress resilience.

Cold stress substantially suppresses plant metabolic activity, leading to marked reductions in chlorophyll biosynthesis, photosynthetic enzyme activity, stomatal conductance, respiration efficiency, and transpiration. These physiological constraints may reduce aerobic respiration efficiency and could potentially favor anaerobic processes under certain conditions, which might lead to localized oxygen limitations within plant tissues (Feng et al. 2025; Li et al. 2025; Liu et al. 2023). Although previous studies mainly described the metabolic and physiological alterations underlying these oxygen-restricted states, the combined effects of impaired gas exchange, restricted oxygen diffusion, and reduced metabolic turnover are consistent with physiological changes that may lead to hypoxia-like states during cold exposure. These conditions may create a physiological context where hypoxia-associated pathways are more likely to be activated during cold stress.

In addition to photosynthetic inhibition, cold stress promotes excessive ROS accumulation, disturbs osmotic homeostasis, reduces root water uptake, and alters hormonal balance, all of which may further influence cellular oxygen status (Feng et al. 2025; Li et al. 2025). These processes contribute to a progressive decline in metabolic rate and oxygen availability, reinforcing the formation of hypoxia-like microenvironments during low-temperature exposure. Stomatal closure and reduced transpiration could limit gas exchange to some extent, which might influence internal oxygen dynamics, although the extent of this effect under cold stress remains uncertain (Li et al. 2025; Liu et al. 2023). These physiological observations collectively support a model in which cold stress inherently generates oxygen-limiting conditions that may trigger hypoxia-responsive transcriptional programs.

Within this physiological background, our transcriptome analysis revealed that cellular response to hypoxia was the most enriched GO-BP category among genes upregulated in *AtBBX11*-overexpressing plants under cold stress (Figure 6). This result aligns with the notion that cold-induced metabolic suppression can activate hypoxia-related signaling pathways. Moreover, several hypoxia-associated genes and transcription factors showed stronger induction in *AtBBX11*-OX plants than in WT plants under cold treatment, as validated by RT-qPCR (Figure 7). These findings suggest that *AtBBX11* overexpression may influence transcriptional responses that help stabilize cellular metabolism during oxygen limitation, although the precise regulatory mechanisms remain to be elucidated. Taken together, the convergence of physiological observations from previous studies and our transcriptomic data supports a model in which *AtBBX11* may contribute to cold tolerance by modulating gene networks responsive to hypoxia-like states induced by low temperature.

To further explore the transcriptional pathways potentially influenced by AtBBX11, we investigated transcription factors among the differentially expressed genes (DEGs) under cold stress. Specifically, we selected transcription factors showing a fold-change greater than 1.5 with *p*≤0.05 in the *AtBBX11*-OX versus wild-type comparison (Table 1). These transcription factors were distributed across several families, with *NAC* (three genes) and *bHLH* (two genes) being the most represented, while the remaining families were each represented by a single gene. Members of the *NAC* family are well known for their involvement in multiple abiotic stress responses, including cold, salinity, drought, and senescence, where they regulate processes such as chloroplast stability, antioxidant defense, and cell cycle–associated gene expression (Han et al. 2023; Shao et al. 2015). Because transcription factors frequently exhibit functional redundancy, we assessed phylogenetic relationships among all selected transcription factor candidates to identify closely related gene pairs that might share overlapping functions. Among the *NAC* family members, *NAC085* and *NAC044* are closely related to each other (Ooka et al. 2003), suggesting possible functional redundancy.

BBX transcription factors are known to interact with various signaling components and to form protein–protein complexes with other regulatory proteins (Bandara et al. 2022; Gangappa and Botto 2014). Such interactions generally enable coordinated control of downstream target genes. Although direct interaction partners of AtBBX11 remain unknown, it is plausible that AtBBX11 may contribute to early cold stress responses through its involvement in regulatory complexes that facilitate transcriptional reprogramming under stress conditions. This possibility requires further experimental validation. Collectively, the transcriptional changes associated with *AtBBX11* overexpression suggest that *AtBBX11* may participate in broader stress-responsive regulatory networks during cold exposure, while its specific contribution to cold tolerance remains to be determined with additional functional analyses.

Given the evolutionary relationships within the *BBX* family, previous phylogenetic analyses indicated that the *BBX* genes most closely related to *AtBBX11* (*COL13*) are *AtBBX12* (*COL14*) and *AtBBX13* (*COL15*), raising the possibility that these proteins may share overlapping functions in regulating gene expression under stress conditions (Khanna et al. 2009). However, Arabidopsis eFP Browser expression data revealed that only *AtBBX13* showed a cold-induced expression pattern similar to *AtBBX11*, whereas *AtBBX12* did not display notable induction under 4°C treatment. This divergence in expression profiles suggests that potential redundancy within this *BBX* subclade may be limited or context dependent. Future studies using Chimeric REpressor gene Silencing Technology (CRES-T) will help clarify whether functional redundancy indeed exists among these *BBX* family members.

Together, our findings indicate that *AtBBX11* expression is strongly induced by cold stress and that overexpression of *AtBBX11* enhances chilling tolerance in Arabidopsis. Transcriptome analysis further revealed that numerous stress-responsive genes were upregulated in *AtBBX11*-overexpressing plants under cold conditions, with cellular response to hypoxia emerging as the most enriched GO category. These results suggest that the enhanced cold tolerance observed in *AtBBX11* overexpression lines may involve, at least in part, transcriptional pathways associated with hypoxia-related responses. While the precise mechanisms remain unclear, our study highlights AtBBX11 as a potential regulator linking cold stress signaling with hypoxia-associated gene expression. Future work integrating molecular, biochemical, and genetic approaches will be essential to clarify how AtBBX11 influences these pathways and to determine its specific role within the broader regulatory network underlying plant cold stress acclimation.

## Abbreviations

ABA: abscisic acid
BBX: B-box
CK: control
CRES-T: Chimeric REpressor gene Silencing Technology
CS: cold stress
DEG: differentially expressed gene
DEPC: diethyl pyrocarbonate
GO: Gene Ontology
MES: 2-(N-morpholino)ethanesulfonic acid
OX: overexpression
RNA-seq: RNA sequencing
ROS: reactive oxygen species
RT-qPCR: real-time quantitative polymerase chain reaction
SPX: SYG1/Pho81/XPR1 domain
*STO*: *SALT TOLERANCE*
WT: wild-type
*ZFPL*: *Zinc finger protein-like*

## Description of supplementary files

Supplementary Table S1. Expression levels of Arabidopsis *BBX* transcription factor family genes under cold stress based on Arabidopsis eFP Browser data.

Supplementary Table S2. Cold-responsive genes identified among the top 500 genes co-expressed with *AtBBX11.*

Supplementary Table S3. List of primers used in this study.

Supplementary Figure S1. Gene Ontology (GO) Biological Process (BP) enrichment analysis of *AtBBX11* co-expressed genes from ATTED-II database.

Supplementary Figure S2. Comparative GO-BP analysis of DEGs in wild-type Arabidopsis and *AtBBX11*-OX lines under cold treatment.

Supplementary Figure S3. Expression profiles of the selected hypoxia-responsive genes under hypoxia based on the Arabidopsis eFP Browser data.
