# Supplementary material for: Overexpression of Arabidopsis BBX11 confers enhanced cold tolerance and alters stress-responsive transcriptional networks
Source: Plant Biotechnol (Tokyo). 2026 Jun 25;43(2):161–73. doi: 10.5511/plantbiotechnology.26.0109a (PMC13324297; doi:10.5511/plantbiotechnology.26.0109a)
Supplement: Supplementary Data [file plantbiotechnology-43-2-26.0109a-s001.pdf]

Supplementary files

Supplementary Table S1. Expression levels of Arabidopsis *BBX* transcription factor family genes under cold stress based on Arabidopsis eFP Browser data

Supplementary Table S2. Cold-responsive genes identified among the top 500 genes co-expressed with *AtBBX11*

Co-expression ranking	Gene name	Locus	Function
3	LTI30	AT3G50970	Dehydrin family protein, LOW TEMPERATURE-INDUCED 30
16	DREB2C	AT2G40340	Integrase-type DNA-binding superfamily protein
22	GI	AT1G22770	Gigantea protein (GI)
26	ERD7	AT2G17840	Senescence/Dehydration-associated protein-like protein
31	DREB2A	AT5G05410	DRE-binding protein 2A
38	RCI2A	AT3G05880	Low temperature and salt responsive protein family
39	TIL	AT5G58070	Temperature-induced lipocalin
62		AT4G30650	Low temperature and salt responsive protein family
84	COR47	AT1G20440	Cold-regulated 47
91	COR413-PM1	AT2G15970	Cold regulated 413 plasma membrane 1
101	LEA14	AT1G01470	Late embryogenesis abundant protein
113	CCR1	AT4G39260	Cold, circadian rhythm, and RNA binding 1
139	DREB1A (CBF3)	AT4G25480	Dehydration response element B1A
144	COR15B	AT2G42530	Cold regulated 15b
151	GRP7	AT2G21660	Cold, circadian rhythm, and RNA binding 2
177	RD29A, LTI78	AT5G52310	Desiccation-responsive protein 29A (RD29A) / Low-temperature-responsive protein 78 (LTI78)
338		AT4G30660	Low temperature and salt responsive protein family
393	KIN2 (COR6.6)	AT5G15970	Stress-induced protein (KIN2) / Cold-responsive protein (COR6.6)
411	COR15A	AT2G42540	Cold-regulated 15a

This table lists genes identified as related to cold stress among the top 500 genes co-expressed with *AtBBX11*, based on data retrieved from the ATTED-II database (https://atted.jp/; Obayashi et al. 2022). Genes were first ranked by co-expression with *AtBBX11*, and those with known associations to cold stress were included in this table.

Obayashi T, Hibara H, Kagaya Y, Aoki Y, Kinoshita K (2022) ATTED-II v11: a plant gene co expression database using a sample balancing technique by subagging of principal components. *Plant Cell Physiol* 63: 869–881

Supplementary Table S3. List of primers used in this study

Gene name	Primer name	Sequence (5'-3')
AtTIP41	AT4G34270_Rf1	GCAGCACAATGGAAATTCAGG
	AT4G34270_Rr1	GCCTCAACCGTTTCTTTGTC
AtRD29A	AT5G52310_Rf1	TGGACAAAGCAATGAGCATGAGC
	AT5G52310_Rr1	AGGTTTACCTGTTACGCCTGGTG
AtRD29B	AT5G52300_Rf2	CCCACGCATAAAGGTGGAGA
	AT5G52300_Rr2	AACTCATGGCTTCTCGTCGG
AtBBX11	AT2G47890_Rf1	TGCCTAAGAATCGTCACGCCACCT
	AT2G47890_Rr1	TAGTCCACGGAGCTGTCGGATCAT
AtHDG6	AT3G44460_Rf1	TCCCATGACTTGCGTGACTCTG
	AT3G44460_Rr1	CACGTTGACCCATTTGCCTGTG
AtbHLH168	AT1G10586_Rf1	TGCGCATGAAACATCTCTTCTCC
	AT1G10586_Rr1	ATAAGTTGAGGCACTGGCAACC
AtNAC085	AT5G14490_Rf1	CACCCTCAAAACCTTCCAGGTGTG
	AT5G14490_Rr1	TTCTCCGCTTTCTCTGCCCATTTT
AT5G20790	AT5G20790-Rf1	TGCGATGACGGAGTGACAAAGG
	AT5G20790-Rr1	TAACACACCTCCCACCGTCAAC
AtPS2	AT1G73010-Rf1	CATAGCTGCTCTTGTGGCACTTG
	AT1G73010-Rr1	TCCTCTCAATGATCAAACCCTTGC
AtSPX1	AT5G20150-Rf1	TGCCGCCTCTACAGTTAAATGGC
	AT5G20150-Rr1	TGGCTTCTTGCTCCAACAATGG
AtDREB2A	AT5G05410_Rf1	TGTCTGGAGAATGGTGCGGAAG
	AT5G05410_Rr1	TCGCTCAGCCAATGCTTATCCG
AtDREB2C	AT2G40340_Rf1	TGTGGCTCGGTACTTTCTCCAGTT
	AT2G40340_Rr1	CATATATAGCTTTGGCCGCCTCGT
AtCOR15A	AT2G42540_Rf1	AATGCTAACATGAGCTGTTCTCA
	AT2G42540_Rr1	CGCTGTGGAAAGAAGAAGCC
AtCOR15B	AT2G42530_Rf1	CACAACGTAGGAGCAAAGCA
	AT2G42530_Rr1	GAGGATGTTGCCGTCACTTT
AtCOR47	AT1G20440_Rf1	CAGTGTCGGAGAGTGTGGTG
	AT1G20440_Rr1	ACAGCTGGTGAATCCTCTGC
AtCBF3	AT4G25480-Rf1	GGATCATGGCTTCGACATGG
	AT4G25480-Rr1	GCTCTGTTCCGCCGTGTAAA

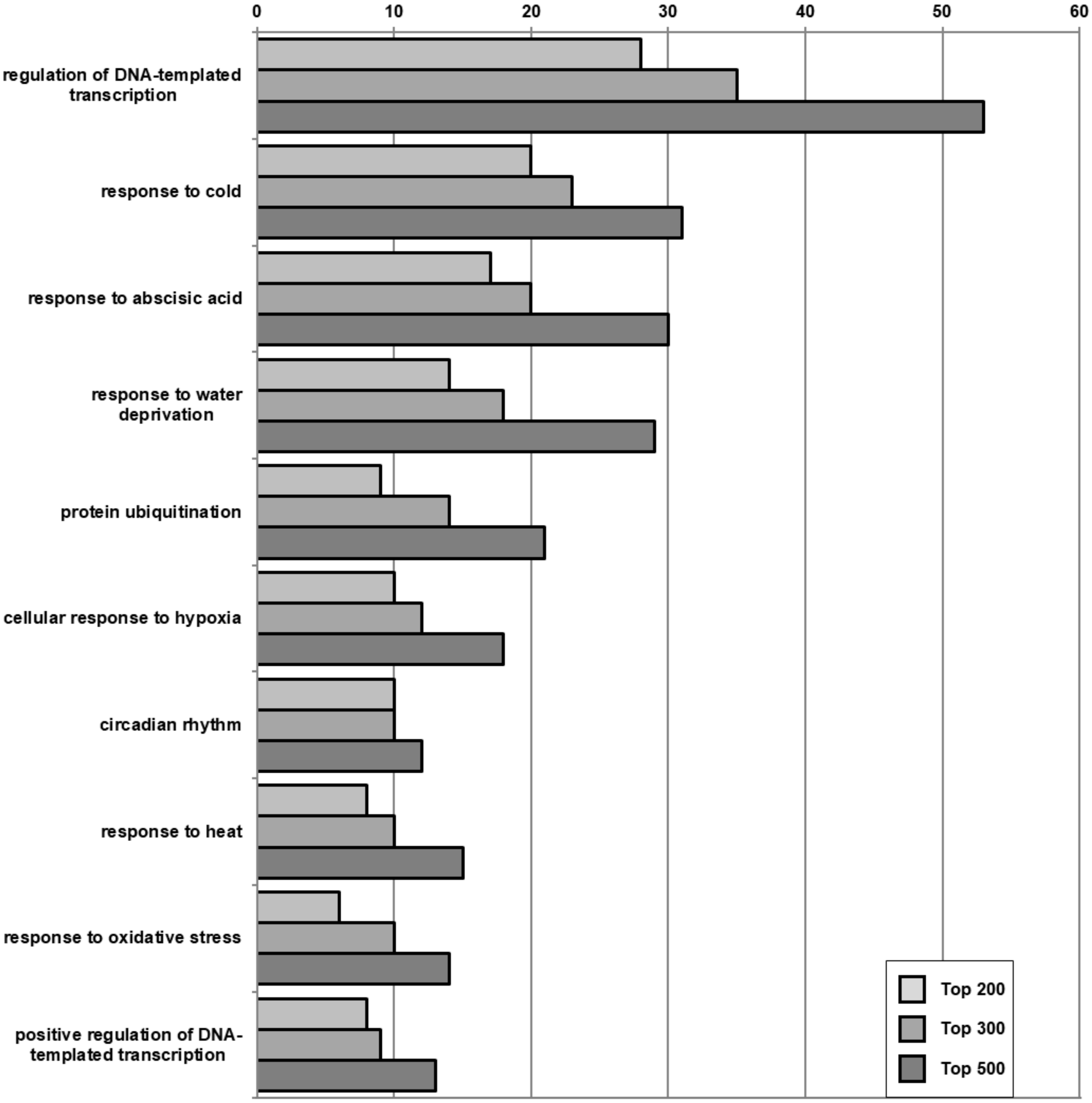

Supplementary Figure S1: Gene Ontology (GO) Biological Process (BP) enrichment analysis of *AtBBX11* co-expressed genes from ATTED-II database. Co-expressed gene lists were retrieved from the ATTED-II database (https://atted.jp/; Obayashi et al. 2022), selecting the top 200, top 300, and top 500 genes based on mutual rank correlation with *AtBBX11*. GO-BP enrichment analysis was performed using the respective gene lists to identify overrepresented biological processes. The figure illustrates the distribution of co-expressed genes across major biological process categories. The X-axis represents the number of genes associated with each GO term, and the Y-axis displays the biological process categories. Bars with different shading patterns correspond to the top 200 (light gray), top 300 (medium gray), and top 500 (dark gray) co-expressed gene datasets, respectively, enabling comparative visualization of enrichment patterns across varying stringency levels.

A

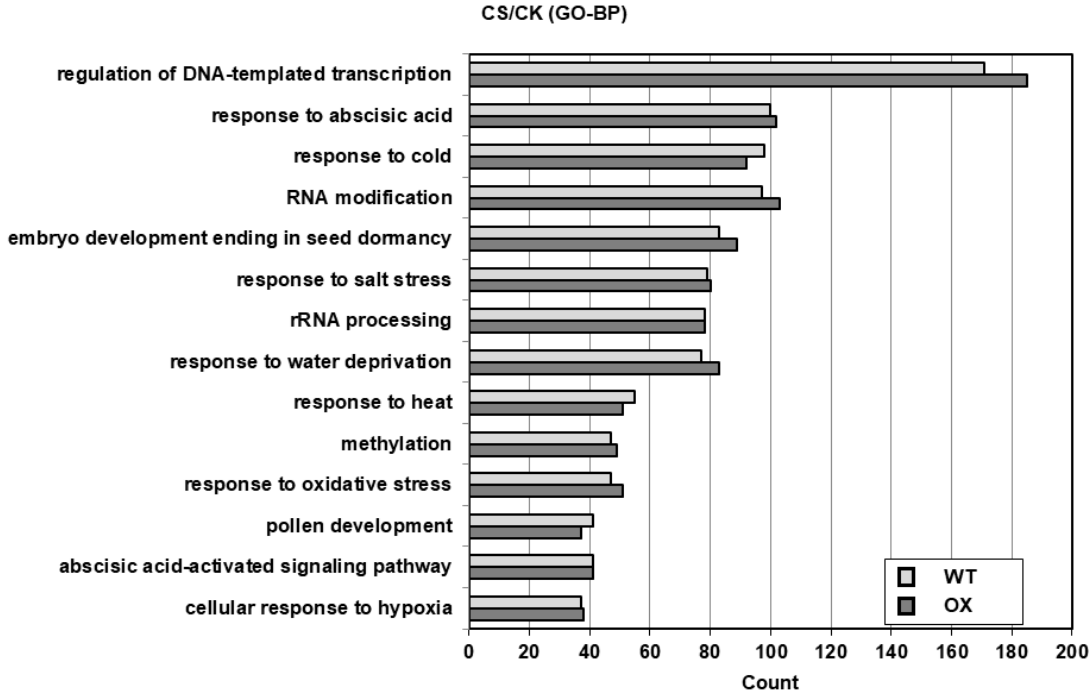

B

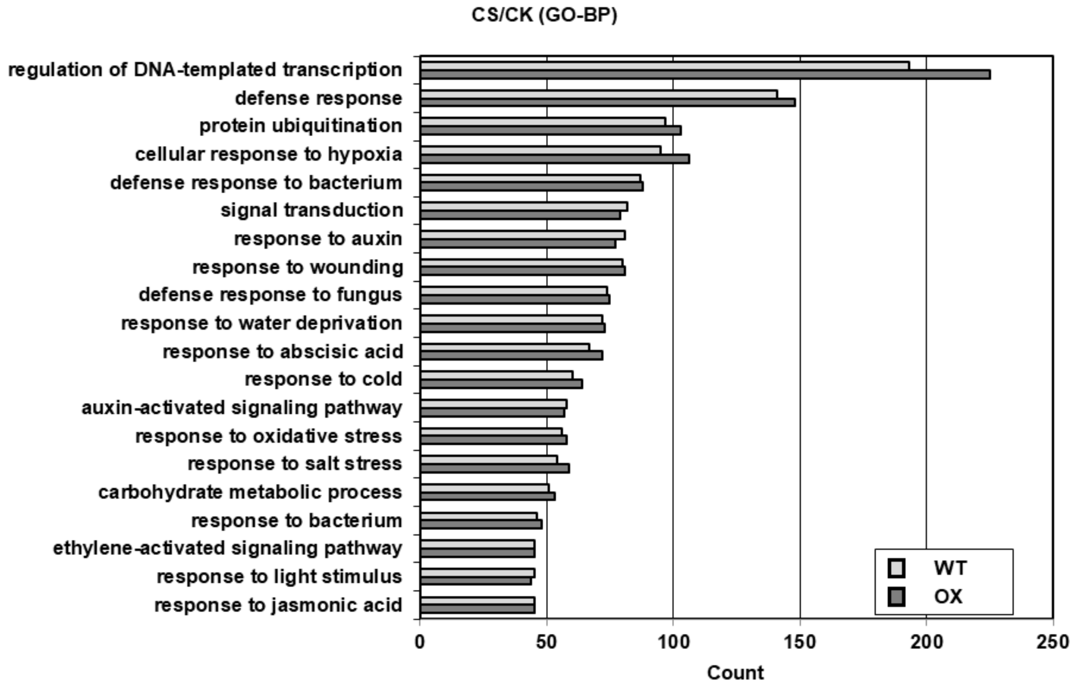

Supplementary Figure S2. Comparative GO-BP analysis of DEGs in wild-type Arabidopsis and *AtBBX11*-OX lines under cold treatment. (A) GO-BP enrichment analysis of up-regulated genes from CS-WT/CK-WT and CS-OX/CK-OX comparisons. (B) GO-BP enrichment analysis of down-regulated genes from the same comparisons. The X-axis represents the number of differentially expressed genes, and the Y-axis indicates biological process categories.

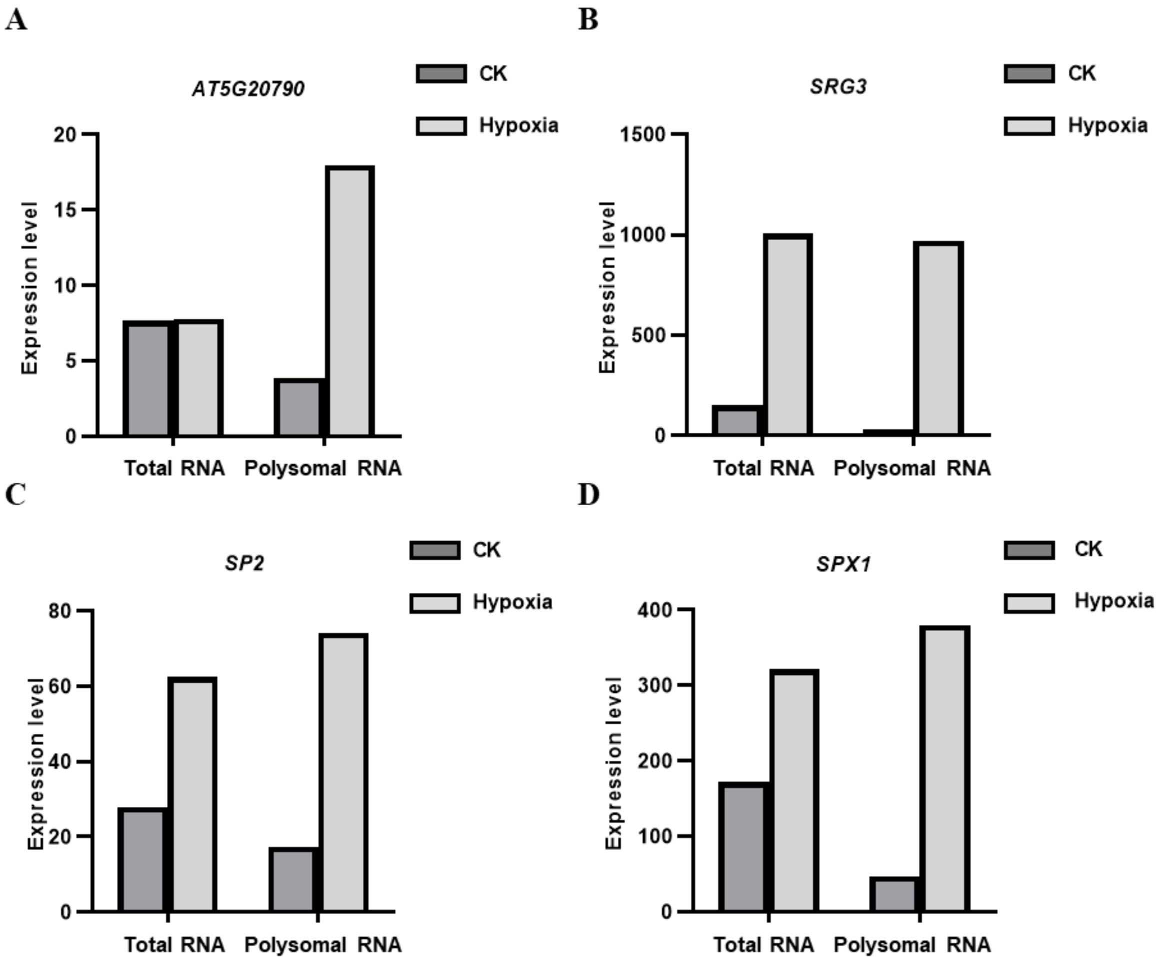

Supplementary Figure S3. Expression profiles of the selected hypoxia-responsive genes under hypoxia based on the Arabidopsis eFP Browser data. Transcript levels of *AT5G20790* (A), *SRG3* (B), *SP2* (C), and *SPX1* (D) in total RNA and polysomal RNA fractions under control (CK) and hypoxia conditions were retrieved from the Arabidopsis eFP Browser database.
